# A nomogram predicting pathological complete response to neoadjuvant chemoradiotherapy for locally advanced rectal cancer: implications for organ preservation strategies

**DOI:** 10.18632/oncotarget.18821

**Published:** 2017-06-28

**Authors:** Yanwu Sun, Pan Chi, Huiming Lin, Xingrong Lu, Ying Huang, Zongbin Xu, Shenghui Huang, Xiaojie Wang

**Affiliations:** ^1^ Department of Colorectal Surgery, Fujian Medical University Union Hospital, Fuzhou, Fujian, PR China

**Keywords:** rectal cancer, nomogram, chemoradiotherapy, pathological complete response

## Abstract

**Purpose:**

To determine predictors of pathological complete response (pCR) in locally advanced rectal cancer patients treated with neoadjuvant chemoradiotherapy (nCRT), and develop a predictive nomogram.

**Methods:**

A total of 522 locally advanced rectal cancer patients undergoing nCRT and curative resection between 2008 and 2014 were included. Uni- and multivariate analysis was performed to identify predictors of pCR. A nomogram was developed and validated by internal (n=425) and external validation (n=97).

**Results:**

With a median follow-up of 55 months, pCR was associated with better 5-year overall and disease-free survival, distant control, but similar local control. Logistic regression showed that post-CRT distance from the anal verge (OR =0.840, P = 0.022), post-CRT tumor size (OR = 0.565, P = 0.003), post-CRT circumferential extent of tumor (OR = 0.021, P < 0.001), pre-CRT CEA level (OR = 2.004, P = 0.033), and post-CRT CEA level (OR = 3.767, P = 0.038) were independently associated with pCR. A nomogram was developed with a C-index of 0.81 and 0.75 on internal and external validation, respectively.

**Conclusion:**

pCR was associated with better long-term outcome. A nomogram was successfully developed to predict pCR. It could support decision-making in organ preservation strategies.

## INTRODUCTION

Neoadjuvant chemoradiotherapy (nCRT) followed by total mesorectal excision (TME) has become the standard of care for locally advanced rectal cancer (LARC), resulting in increased tumor regression and better local control [1, 2]. Approximately 10–30% of LARC patients undergoing nCRT and radical resection will develop pathological complete response (pCR), which is defined as absence of viable tumor cells (ypT0N0M0) in the surgical resection specimen [3–7]. This has translated into not only better local control but also improved overall survival [6, 7].

Radical surgery with TME, however, is associated with significant morbidity, including postoperative complications, urinary and fecal incontinence, sexual dysfunction, and a permanent stoma in some cases [8, 9]. In light of these observations, some surgeons have explored organ preservation strategies—“watch and wait” or local excision (LE), to improve the quality of life of patients achieving pCR [5, 10–12]. Nevertheless, widespread adoption of these novel strategies is limited by the accuracy of identifying which patients would benefit most from the organ preservation approach.

Therefore, reliable prediction of pCR is needed to facilitate tailoring treatment strategies without compromising long-term survival. Efforts have been made to identify possible predictors of pCR, but a reliable method is still lacking. A nomogram is a useful tool for predicting oncological outcomes in various malignancies [13–15]. It could also be developed to predict tumor response to nCRT in patients with LARC [16, 17]. Based on this information, a predictive nomogram might help better identify patients who might present a pCR. Nevertheless, to our knowledge, studies focused on this issue are limited [17, 18].

The aim of this study was to identify, in a large series of patients, post-CRT clinicopathologic and treatment-related factors that predict pCR following nCRT, and to develop a predictive nomogram for pCR.

## RESULTS

### Patients’ characteristics and survival

**Figure 1:**
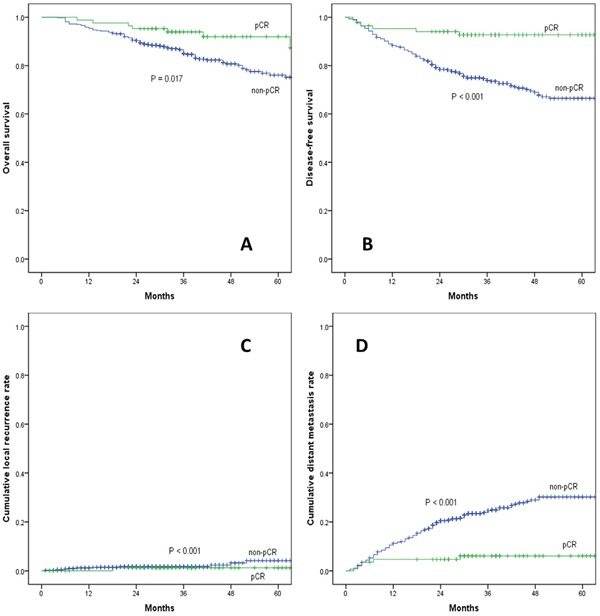
**(A)** overall survival, **(B)** disease-free survival, **(C)** cumulative local recurrence, **(D)** cumulative distant metastasis rate between pCR and non-pCR group. pCR: pathological complete response.

A total of 522 LARC patients were included in this study. Eighty five (85/522, 16.2%) patients experienced pCR after nCRT and TME. With a median follow-up of 55 months (ranging 20-102 months), the 5-year overall survival (pCR vs. non-pCR: 92.0% vs. 76.1%; P = 0.017) and disease-free survival rate (92.7% vs. 66.5%; P < 0.001) were better in the pCR group than in the non-pCR group, as presented in Figure 1. The 5-year local recurrence rate was lower in the pCR group, but the difference was not significant (pCR vs. non-pCR: 1.2% vs. 4.2%; P = 0.380). Additionally, the 5-year distant metastasis rate was significantly lower in the pCR group (pCR vs. non-pCR: 6.1% vs. 31.2%; P < 0.001).

### Independent prognostic factors of pCR

Patients treated with nCRT and TME were divided into the nomogram training (n=425) and validation (n=97) cohort according to the treatment time. No significant differences were found between the two groups in terms of age, sex, ASA scores, distance from the anal verge, gross type, histopathology, tumour differentiation, clinical T stage, clinical N stage, pretreatment CEA levels, surgical approach, and surgical procedure (Table 1). pCR rate in the training group was 17.4% (74/425), slightly higher than 11.3% (11/97) in the validation group, but this difference did not reach statistical significance (P = 0.144).

**Table 1:** Baseline characteristics of training and validation groups

Variables	Training group (n=425)	Validation group (n=97)	P value
Sex (%)			0.650
Male	270 (63.5)	64 (66.0)	
Female	155 (36.5)	33 (34.0)	
Age (years)	55.3 ± 11.7	52.5 ± 13.8	0.064
Distance from the anal verge (cm)	5.8 ± 2.0	5.5 ± 1.8	0.075
Tumour differentiation (%)			0.205
Well or moderately differentiated	352 (82.8)	75 (77.3)	
Poorly differentiated and others ^a^	73 (17.2)	22 (22.7)	
Histopathology (%)			0.093
Adenocarcinoma	377(88.7)	80 (82.5)	
Mucinous or signet ring adenocarcinoma	48 (11.3)	17 (17.5)	
Pretreatment CEA level (%)			0.209
≤5 ng/ml	242 (56.9)	62 (63.9)	
>5 ng/ml	183 (43.1)	35 (36.1)	
Clinical T stage (%)			0.955
T3	104 (24.5)	24 (24.7)	
T4	321 (75.5)	73 (75.3)	
Clinical N stage (%)			0.679
N0	34 (8.0)	9 (9.3)	
N+	391 (92.0)	88 (90.7)	
ypTNM stage (%)			0.015
0	74 (17.4)	11 (11.3)	
I	114 (26.8)	15 (15.5)	
II	113 (26.6)	37 (38.1)	
III	124 (29.2)	34 (35.1)	
pCR(%)	74 (17.4)	11 (11.3)	0.144

Data are expressed as number (%) or as median ± standard deviation, where appropriate. CEA: carcinoembryonic antigen; ypTNM stage: post-chemoradiotherapy pathological TNM stage; pCR: pathological complete response.

^a^ including mucinous and signet cell carcinoma.

On univariate analysis, post-CRT distance from the anal verge (P = 0.005), post-CRT tumor size (P < 0.001), post-CRT circumferential extent of tumor (P < 0.001), tumor pathology (P < 0.001), pre-CRT CEA level (P = 0.001), and post-CRT CEA level (P < 0.001) were independently associated with pCR in LARC patients treated with nCRT and TME (Table 2). When applied to Logistic analysis, post-CRT distance from the anal verge (OR =0.840, 95% CI: 0.723-0.975, P = 0.022), post-CRT tumor size (OR = 0.565, 95% CI: 0.386-0.827, P = 0.003), post-CRT circumferential extent of tumor (OR = 0.021, 95% CI: 0.004-0.114, P < 0.001), pre-CRT CEA level (OR = 2.004, 95% CI: 1.058-3.798, P = 0.033), and post-CRT CEA level (OR = 3.767, 95% CI: 1.080-13.148, P = 0.038) were found to be independently associated with pCR following nCRT (Table 3).

**Table 2:** Univariate analysis of factors associated with pCR in the training cohort (n=425)

Variables	pCR (n=74)	non-pCR (n=351)	P value
Gender (%)			0.286
Male	43 (58.1)	227 (64.7)	
Female	31 (41.9)	124 (35.3)	
Age (years)	55.1±10.7	55.4±11.7	0.821
ASA score (%)			0.295
1	48 (64.9)	232 (66.1)	
2	26 (35.1)	109 (31.1)	
3	0	10 (2.8)	
BMI (kg/m^2^)	22.6±3.2	22.6±3.4	0.990
Post-CRT DAV (cm)	5.2±1.7	5.9±2.1	0.005
Post-CRT tumor size (cm)	2.6±0.9	3.3±1.0	<0.001
Post-CRT circumference of tumor extent	0.38±0.14	0.59±0.26	<0.001
Pre-CRT MRI T stage (%)			0.080
T3	24 (32.4)	80 (22.8)	
T4	50 (67.6)	271 (77.2)	
Pre-CRT MRI N stage (%)			0.327
N0	8 (10.8)	26 (7.4)	
N+	66 (89.2)	325 (92.6)	
Post-CRT MRI T stage (%)			0.178
T0-2	35(47.3)	190(54.1)	
T3	22(29.4)	70(19.9)	
T4	17(23.0)	91(25.9)	
Post-CRT MRI N stage (%)			0.092
N0	55(74.3)	225(64.1)	
N+	19(25.7)	126(35.9)	
Gross type (%)			0.286
Expanding	22 (29.7)	77 (21.9)	
Ulcering	47 (63.5)	255 (72.6)	
Infiltrating	5 (6.8)	19 (5.4)	
Histopathology (%)			0.027*
Adenocarcinoma	71 (95.9)	306 (87.2)	
Mucinous or signet ring adenocarcinoma	3 (4.1)	45 (12.8)	
Tumor differentiation (%)			0.059
Well moderately differentiated	67 (90.5)	286 (81.5)	
Poorly differentiated and others^a^	7 (9.5)	65 (18.5)	
Pre-CRT CEA (%)			0.001
≤5 ng/ml	55 (74.3)	187 (53.3)	
>5 ng/ml	19 (25.7)	164 (46.7)	
Post-CRT CEA (%)			<0.001
≤5 ng/ml	71 (95.9)	276 (78.6)	
>5 ng/ml	3 (4.1)	75 (21.4)	
Radiation dose(cGy)	4936.5±190.0	4888.2±305.9	0.081
Chemotherapy modality (%)			0.185
Fluoropyrimidine only	22 (29.7)	133 (37.9)	
Oxaliplatin based	52 (70.3)	218 (62.1)	
Interval to surgery (weeks)	8.0±1.4	8.0±2.5	0.930
Approach method (%)			0.093
Open	20 (27.0)	131 (37.3)	
Laparoscopy	54 (73.0)	220 (62.7)	
Surgical procedure (%)			0.284
LAR	67 (90.5)	292 (83.2)	
APR	6 (8.1)	50 (14.2)	
Hartmann’s procedure	1 (1.4)	9 (2.6)	

Data are expressed as number (%) or as median ± standard deviation, where appropriate.

pCR: pathological complete response; ASA: American Society of Anesthesiology; BMI: body mass index; CRT: chemoradiotherapy; DAV: distance from the anal verge; CEA: carcinoembryonic antigen; LAR: low anterior resection; APR: abdominoperineal resection.

^a^ including mucinous and signet cell carcinoma.

* Fisher’s exact test.

**Table 3:** Multivariate analysis of factors associated with pCR in the training cohort (n=425)

Variables	Logistic regression				Nomogram	
	Training group (n=425)					
	Regression coefficient	SE	OR(95% CI)	*P* value	C-index	95%CI
Post-CRT DAV, cm	−0.175	0.076	0.840 (0.723-0.975)	0.022		
Pre-CRT CEA level, ≤5 ng/ml	0.695	0.326	2.004 (1.058-3.798)	0.033		
Post-CRT CEA level, ≤5 ng/ml	1.326	0.638	3.767 (1.080-13.148)	0.038	Training: 0.81	0.76-0.85
Tumor pathology, mucinous or signet ring adenocarcinoma	0.532	1.147	1.703 (0.628-7.884)	0.642	Validation: 0.75	0.59- 0.92
Post-CRT tumor, cm size	−0.571	0.194	0.565 (0.386-0.827)	0.003		
Post-CRT circumferential of tumor extent	−3.861	0.861	0.021 (0.004-0.114)	<0.001		

Abbreviations: SE: Standard error; OR, odds ratio; CI, confidence interval; CRT: chemoradiotherapy; DAV: distance from the anal verge; CEA, carcinoembryonic antigen.

### Nomogram for pCR

**Figure 2:**
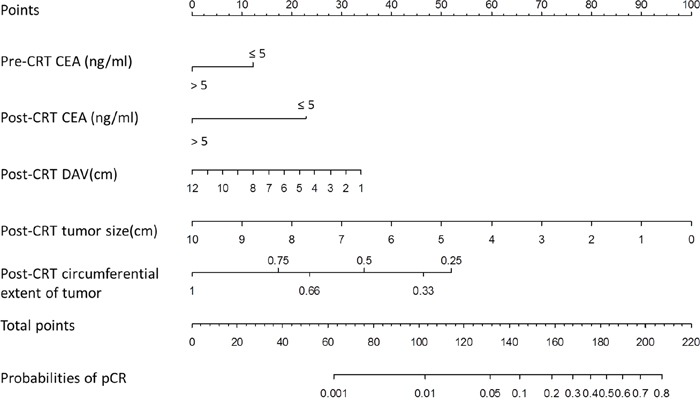
Nomogram developed for prediction of pCR A score for each predictor can be read out at the top scale (score), and the sum of scores is converted to a probability. pCR: pathological complete response DAV: distance of the tumor from the anal verge, CEA: carcinoembryonic antigen.

A nomogram incorporating significant predictors in the Logistic analysis was established to predict pCR in LARC patients following nCRT, as showed in Figure 2. Each subtype within these variables was assigned a score on the point scale. After adding up the total score, a vertical line could be drawn downwards from the total point scale to obtain the probability of pCR (see the bottom scale).

### Validation for nomogram

**Figure 3:**
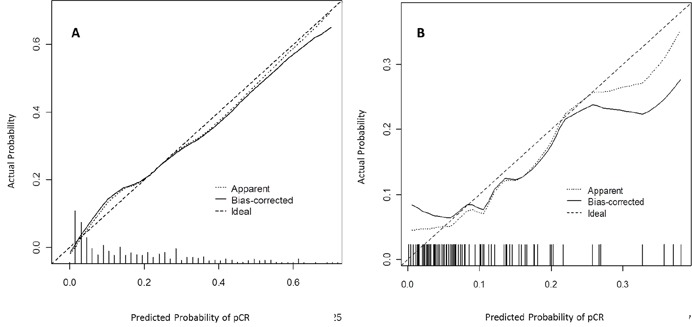
Calibration plots in the internal **(A)** and external **(B)** validation cohort for pCR. The solid line represents the performance of the present nomogram, and the dashed line represents the performance of an ideal nomogram. pCR: pathological complete response.

The nomogram went through two validation procedures: internal (n=425) and external (n=97) validation. The predictive accuracy (measured by C-index) of the nomogram for predicting pCR was 0.81 (95% CI, 0.76 to 0.85). The calibration plots (Figure 3A) presented good statistical performance upon internal validation between the nomogram-predicted probabilities and actual observations of pCR rates. In the external validation cohort, the C-index of the model was 0.75 (95% CI, 0.59 to 0.92), and the calibration curve (Figure 3B) showed the relationship between prediction and observation in the probabilities of pCR rates.

## DISCUSSION

Nowadays, less-invasive organ preservation strategies following nCRT are becoming increasingly popular, and these strategies require accurate prediction of pCR to guarantee oncological outcome. In the present study with a large number of patients, we successfully developed a predictive nomogram for pCR in rectal cancers by including post-CRT clinical factors. Meanwhile, by informing patients the likelihood of pCR, patients can potentially become more actively involved in the decision-making process with regard to organ preservation strategies.

Pathological complete response has been used as a surrogate endpoint for early efficacy and long-term survival in LARC following nCRT. A pooled analysis of 3105 patients from 14 studies has suggested pCR was associated with significantly improved disease-free and overall survivals [4], which was in accordance with our present study. The major implication of pCR to nCRT is that these patients may become eligible for organ preservation strategies. The 5-year LR rate of 1.2% in our series suggested that pCR almost eradicate the risk of LR and organ preservation strategies might be feasible for such patients. However, the effect of pCR on distant failure after surgical excision was not that dramatic. The 5-year DM rate of 6.1%, despite achieving pCR, may be an indication that adjuvant chemotherapy is warranted in these patients.

There is a large variability in tumor response to neoadjuvant treatment among rectal cancer patients [19]. The lack of standardization in patient selection for organ preservation strategies was a confounding factor in previous studies. When local excision or “watch-and-wait” strategies are being considered, patients must undergo a cautious evaluation, including clinical, endoscopic, and radiological assessment, to eliminate the risk of residual tumor. Additionally, an accurate prediction of pCR will provide useful information to assist decision-making in organ preservation strategies. Recently, nomograms have been developed to predict tumor response in LARC patients following nCRT [17, 18]. Studies have identified a variety of disease- and treatment-related variables as potential predictors of pCR. Factors such as tumor characteristics, combined with clinical, serologic and imaging parameters might allow for the development of a nomogram that can predict pCR with a good sensitivity and specificity.

CEA has been widely used to predict the response to neoadjuvant treatment in LARC patients. Pre-CRT CEA levels are indicative of tumor aggressiveness, and post-CRT levels might be an indicator of both tumor aggressiveness and a response to CRT. Additionally, tumor cells containing a high density of CEA may be resistant to radiation [20]. A recent study by Probst et al [21] demonstrated that rectal cancer patients with elevated pretreatment CEA are less likely to experience pCR, pathological tumor regression and downstaging, suggesting that these patients may not be suitable for “watch-and-wait” strategy. Perez et al. [22] determined that post-CRT CEA levels <5 ng/ml was a favorable prognostic factor for rectal cancer and was predictive of pCR. Another recent study by Kleiman et al. [23] showed that post-CRT CEA levels were significantly lower in LARC patients with pCR (1.7 vs. 2.4 mg/L, P<0.01), indicating that the normalization of post-CRT CEA levels was a strong predictor of achieving pCR. Similarly, we found that low pre-CRT and/or post-CRT CEA levels (≤5 ng/ml) were significant predictors of pCR, which was in accordance with previous studies. Although the exact mechanism is unclear and has yet to be elucidated, we suppose that the lower post-CRT CEA level implies a lower tumor burden and, subsequently, less residual tumor in the rectum after CRT. From our results, by incorporating both pre- and post-CRT CEA into the predictive nomogram, it could provide information about CEA change during neoadjuvant treatment, and thus more individualized information for the prediction of pCR.

Smaller tumor size has been found to be the most common factor related to an increased rate of pCR, suggesting that this variable should be considered when risk-stratifying patients for the “watch-and-wait” approach [24, 25]. In addition, two randomized clinical trials have reported that reduction of the tumor to 2-3 cm was usually required for patients to be qualified for local excision after nCRT [26, 27]. The present study also showed that larger post-CRT tumor size predicted lower pCR following nCRT, which was in accordance with previous studies. Nevertheless, the true effect-size significance could be questionable, due to the different scale used to stratified post-treatment lesion. Although we looked for different cut-offs, results remained comparable: greater tumor size predicts lower pCR.

Other disease-related variables, such as circumferential tumor extent and distance of tumor from the anal verge, are also important in prediction of pCR. Das et al [28] evaluated predictors of pCR in 562 rectal cancer patients and found that the circumferential tumor extent and distance of tumor from the anal verge were predictive of pCR. Yan et al [29] have demonstrated that tumor circumferential extent >50% was significantly associated with a poor pathologic tumor response. Similarly, we demonstrated lesser circumferential extent of tumor and lower distance from the anal verge was significantly associated with pCR.

Several studies have also found a number of treatment-related variables that are associated with pCR, including the interval to surgery, radiation dose, and chemotherapy regimen [30–32]. In our study, neither the dose of neoadjuvant radiation, nor the time interval to surgery, was correlated with pCR. One of the explanations may be that surgery was generally performed 6–8 weeks after the completion of nCRT, and patients in this period received the consistent treatment strategies, including nCRT and surgery.

The facility to predict pCR may allow selective application of an organ preservation strategy in the preoperative setting. The present nomogram basing on clinical parameters has reliable C-index on internal validation; however, the discriminative ability was reduced in external validation. The small sample size and some missing data in external validation cohort might be the main contributor to this. Adding specific molecular markers and genetic signatures into the prediction model might increase model accuracy.

There are some potential limitations that warrant discussion. First, this nomogram is based on a retrospective analysis from a single institution. Its applicability to the general population requires further external validation from multiple institutions. Nevertheless, patients in our series received the same pretreatment work-up, postoperative treatment and surveillance strategies, indicating that this nomogram is a reliable reference that can be employed to further investigation. Second, patients who treated with local excision (n=10) or non-operative management (n=3) were excluded from this study, and it might be a source of potential bias. Nevertheless, histological assessment of a TME specimen is considered the gold standard method for determining pCR. Inclusion of such patients in the analysis would lead to the lack of nodal staging information for these patients. Another limitation is that some information, such as endoscopic evaluation, fluorine-18-fluorodeoxyglucose positron emission tomography/computed tomography (18F-FDG PET/CT) findings and gene expression profiling, was not available for all patients, and thus was not evaluated in this study. Despite these limitations, we hope that our experience will contribute to accurately predict pCR. Future work will focus on validating this model, both on external validation from other institutions and incorporation of other predictors to the model.

In conclusion, pCR was related to improved long-term outcome in LARC. This large, retrospective study identified post-CRT clinical parameters, such as distance from the anal verge, tumor size, circumferential extent of tumor, and post-CRT CEA level, as predictors of pCR in LARC patients following nCRT. The predictive nomogram could help physicians predict pCR, and support decision-making in organ preservation strategies. Further studies in a larger series are warranted to validate these results.

## PATIENTS AND METHODS

### Patient population

Based on our prospectively maintained database, we identified 522 consecutive LARC patients who underwent nCRT and curative resection between 2008 and 2014. The inclusion criteria were as follows: (1) clinical stage II or III, (2) tumors located within 12 cm from the anal verge, (3) a histologically proven adenocarcinoma. The exclusion criteria were as follows: (1) previous or concurrent malignancies, (2) patients treated with emergent surgery, palliative resection, and (3) patients treated with local excision or “watch and wait” strategy. Our institutional review board approved this study. Patients treated with nCRT and TME were divided into the nomogram training (n=425) and validation (n=97) cohort according to the treatment time, that is, from 2011 to 2014 and from 2008 to 2010.

### Treatment

Patients were staged and restaged by the same surgical team using digital rectal examination, abdominopelvic magnetic resonance imaging (MRI) and/or transrectal ultrasonography (ERUS). Generally, nCRT was more often offered to those with more advanced rectal cancers (T4 and/or N+), and tumor required downsizing for clear surgical margins or sphincter preservation. Nevertheless, the final decision was made by the patients based on the current stage of their disease and after understanding the risks and benefits and without the influence of the surgeons. Preoperative long-course radiotherapy protocol consisted of a total dose of 50.4 Gy delivered in fractions of 1.8 Gy with 5 fractions per week for 5 weeks followed by a boost of 5.4 Gy. Preoperative chemotherapy was administered concurrently with radiotherapy using two regimens: 5-FU plus oxaliplatin (FOLFOX) and capecitabine plus oxaliplatin (CapeOX).

Surgery was performed 6-8 weeks after the radiation was completed. Surgical techniques for rectal cancer, such as TME and high ligation of the inferior mesenteric artery, were standardized at our institution. TME was performed for middle and low rectal cancers, and partial TME with a distal margin of 5cm was performed for high rectal cancers. Starting approximately 3 to 4 weeks after surgery, patients received adjuvant chemotherapy for 6 months. Two different chemotherapy regimens were used, FOLFOX and CapeOX.

Patient follow-up was scheduled for every 3 months for the first 3 years, then every 6 months for the next 2 years, and annually thereafter. Physical examination, serum carcinoembryonic antigen (CEA) level, chest X-ray or CT, and abdominopelvic MRI or CT scans were performed at each visit. A colonoscopy was conducted annually after surgery. Positron emission tomography (PET) was performed when needed. Patient follow-up lasted until death or until the cut-off date of August 30, 2016.

### Pathological examinations

**Figure 4:**
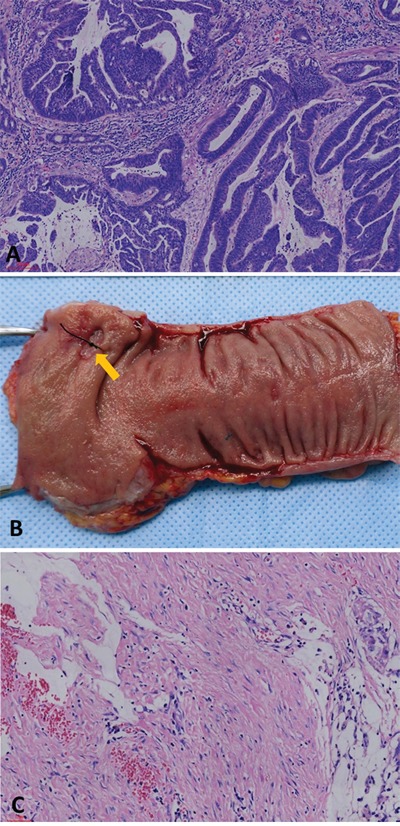
**(A)** Moderately differentiated adenocarcinoma in biopsied specimen before nCRT (hematoxylin and eosin stain, 100×). **(B)** No apparent tumor but an ulcer (arrow) in the resected specimen after nCRT. **(C)** No residual adenocarcinoma was found in the original ulcerated adenocarcinoma site. Instead, it was completely replaced by dense fibrous tissue, infiltration of lymphocytes and macrophage, and acellular mucin “lake”.

Pathologic complete response was defined as absence of any viable adenocarcinoma cells in the resection specimen, including the bowel wall or regional lymph nodes (ypT0N0M0), as showed in Figure 4. A pCR was diagnosed by at least two experienced pathologists, and tissue blocks were taken from the entire tumor site to confirm the absence of viable tumor cells.

### Definitions

Tumor size (based on its maximum diameter) and circumferential extent of tumor were assessed by ERUS after nCRT or, when this was not available, by rigid sigmoidoscopy. Tumor distance from the anal verge was assessed after nCRT using rigid proctoscopy with insufflation by the same surgical team. Local recurrence (LR) was defined as any tumor relapse within the pelvis, perineum, or anastomosis as diagnosed by imaging and/or pathological examination. Distant metastasis (DM) was identified as evidence of a tumor in any other area diagnosed by imagining or pathological examinations.

### Statistical analysis

Statistical analysis was performed using SPSS version 20.0 (SPSS INC., Chicago). Univariate analysis was performed using the Chi-square test or Fisher’s exact test for categorical variables and Student’s t-test for continuous variables. All significant variables on univariate analysis were entered into a logistic regression model to identify predictors of pCR. Based on the multivariable analysis, a nomogram was formulated by using R 2.12.1 (The R Foundation for Statistical Computing, Vienna, Austria). The performance of nomogram was evaluated by calculating the Harrell’s concordance index (C-index). The nomogram went through two validation procedures: internal and external validation. Calibration of the nomogram for pCR rates was performed by comparing the predicted probability and the actual status after bias correction. Statistical significance was accepted at P < 0.05.

## Abbreviations

nCRT: neoadjuvant chemoradiotherapy
TME: total mesorectal excision
LARC: locally advanced rectal cancer
pCR: pathological complete response
MRI: magnetic resonance imaging
ERUS: endorectal ultrasounography
LE: local excision
RCRG: Rectal Cancer Regression Grade
LR: local recurrence
DM: distant metastasis
18F-FDG PET/CT: fluorine-18- fluorodeoxyglucose positron emission tomography/computed tomography
